# Synthesis of Chiral Chalcone Derivatives Catalyzed by the Chiral Cinchona Alkaloid Squaramide

**DOI:** 10.3390/molecules191219491

**Published:** 2014-11-25

**Authors:** Dandan Xie, Ying Xie, Yan Ding, Jian Wu, Deyu Hu

**Affiliations:** State Key Laboratory Breeding Base of Green Pesticide and Agricultural Bioengineering, Key Laboratory of Green Pesticide and Agricultural Bioengineering, Ministry of Education, Guizhou University, Huaxi District, Guiyang 550025, China; E-Mails: xddxed@163.com (D.X.); xieying2045@163.com (Y.X.); diding1011@163.com (Y.D.); wujian2691@126.com (J.W.)

**Keywords:** chalcone derivatives, enantioselective catalysis, chiral cinchona alkaloidsquaramides, addition reaction

## Abstract

An effective method has been developed for the preparation of novel chiral chalcone derivatives under mild conditions from the easily accessible starting materials nitromethane and chalcone derivatives **2**. The corresponding products were obtained in moderate yields with excellent enantioselectivities (up to 99%).

## 1. Introduction

**Figure 1:**
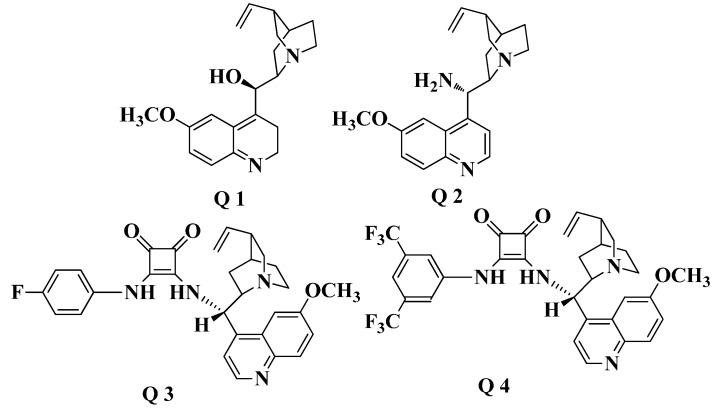
The catalysts surveyed in the work.

**Scheme 1:**
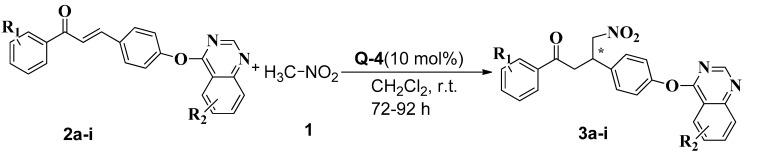
Synthesis of Chiral Chalcone Derivatives **3a–i**.

Chalcones are not only excellent scaffolds for synthetic manipulations but also possess multiple biological and medicinal properties as antioxidant [1], antibacterial [2,3,4], antifungal [5], anti-Alzheimer’s disease [6], anticancer [7], antitumor [8], antimalarial [9], antiproliferative [10], anti-inflammatory [11], and anti-HIV-1 agents [12]. Research laboratories worldwide are focusing on the synthesis of different chalcone analogues for the development of novel and potent drugs [13]. Asymmetric catalysis has proven its potential in numerous demanding applications which have been reported and developed for the direct asymmetric addition reactions of chalcones in the past. Lu *et al*. reported a highly diastereoselective and enantioselective conjugate addition of phthalide derivatives to chalcones, leading to the formation of chiral phthalides bearing vicinal quaternary and tertiary stereogenic centers [14]. Previous work using chiral squaramide-based organ catalysts in similar enantioselective Michael additions have been reported [15,16], and asymmetric cyclopropanation of chalcones using bromomalonates as the nucleophiles in a Michael-Initiated Ring Closing reaction was developed by Waser [17]. The reaction performed well for electron neutral and electron deficient chalcones, giving the products in yields of up to 98% and with enantiomeric ratios up to 91:9. Surprisingly, the use of chiral squaramide catalysts and nitromethane to facilitate addition reactions was described only a few times. Based on this concept and the knowledge gathered therein we surmised that some catalyst structures Q1–Q4 might be efficient in the direct addition reactions between nitromethane (**1**) with chalcone derivatives **2** (Figure 1). Herein we described our work toward preparation of such interesting chiral moieties in the presence of a variety of known chiral cinchona alkaloid squaramides (Scheme 1).

## 2. Results and Discussion

### 2.1. Chiral Catalysts Screening

As shown in Figure 1, various catalysts including cinchona alkaloids Q1 and Q2, and cinchona alkaloid-derived squaramides Q3 and Q4 were developed and surveyed in the reaction. All the catalysts surveyed in the work ubiquitously formed the chiral product **3g** (R_1_ = 4-Cl, R_2_= 6-Me). The results are summarized in Table 1. (*E*)-1-(4-Chlorophenyl)-3-(4-((6-methylquinazolin-4-yl)oxy)phenyl) prop-2-en-1-one (**2g**) and nitromethane (**1**) were adopted as the starting materials for the initial exploration of the asymmetric addition reaction. The reaction was carried out in dichloromethane in the presence of 10 mol % catalyst at room temperature for 72 h. Using catalyst Q1, the product **3g** was obtained in 60% *ee* (Table 1, entry 1). As for the other catalysts, Q2 and Q3 afforded lower yield and moderate enantioselectivity (Table 1, entry 2 and entry 3, 45% and 70% *ee*, respectively) in the same solvent. Higher *ee* values were obtained in dichloromethane with catalyst Q4 (Table 1, entries 4, 96%). Cinchona alkaloid-derived squaramide Q4 gave excellent enantioselectivity with moderate yield.

**Table 1:** Catalyst screening studies ^[a]^.

Entry	Catalyst	Solvent	Yield [%] ^[^^b^^]^	*ee* [%] ^[^^c^^]^
1	Q1	CH_2_Cl_2_	20.2	60
2	Q2	CH_2_Cl_2_	35.0	45
3	Q3	CH_2_Cl_2_	37.2	70
4	Q4	CH_2_Cl_2_	46.0	96

^[a]^ Unless otherwise indicated, all reactions were conducted with **1** (0.9 mmol), **2g** (0.45 mmol) and the catalyst (10 mol %, 0.045 mmol) in dichloromethane (4 mL) at room temperature for 72 h. ^[b]^ Isolated yield after chromatographic purification. ^[c]^ Determined by HPLC analysis (ChiralpakAD-H).

**Table 2:** Optimization studies ^[a]^.

Entry	Temperature [°C]	Solvent	Yield [%] ^[^^b^^]^	*ee* [%] ^[^^c^^]^
1	rt	CH_2_Cl_2_	46.2	96
2	rt	EtOH	30.0	50
3	rt	PhCH_3_	40.9	67
4	60	PhCH_3_	60.2	55
5	0	CH_2_Cl_2_	46.0	79
6	40	CH_2_Cl_2_	50.2	85
7	rt ^[d]^	CH_2_Cl_2_	40.2	88
8	rt ^[e]^	CH_2_Cl_2_	55.2	97

^[a]^ Unless otherwise indicated, all reactions were conducted with **1** (0.9 mmol), **2g** (0.45 mmol) and Q4 catalyst (10 mol-%, 0.045 mmol) in the different solvents (4 mL) at different temperatures for 72 h. ^[b]^ Isolated yield after chromatographic purification. ^[c]^ Determined by HPLC analysis (Chiral pakAD-H). ^[^^d]^ Reaction time: 42 h. ^[^^e]^ Reaction time: 100 h.

### 2.2. Optimization Studies

To optimize the reaction regime, the synthesis of chiral compound (−)-**3g** was carried out under several conditions. The effects of different solvents, reaction time and temperature were investigated using Q4 as catalyst; the results are shown in Table 2. From the data presented in the table it can be concluded that dichloromethane appeared to be the best solvent to obtain high *ee* values; other solvents provided much poorer enantioselectivities ranging between 50%–67% *ee* (Table 2, entry 1, entry 2 and entry 3). Moreover, the effects of reaction temperature and time on the addition reaction were also investigated (Table 2, entries 5–7). When the reaction time was prolonged from 42 to 72 h, the *ee* value of (−)-**3g** was increased from 88% to 96% (Table 2, entries 4 and 6). Extending the reaction time up to 100 h resulted in only a tiny improvement of the *ee* value (97%, Table 2, entry 8) if compared with that obtained after 72 h of reaction (96%, Table 2, entry 4). As for the reaction temperature, it could be observed that when the reaction temperature was increased from 0 °C to room temperature and 40 °C, the *ee* values of (−)-**3g** were 79%, 96% and 85%, respectively (Table 2, entries 1, 5–6). Hence, the optimal reaction conditions were selected as following: CH_2_Cl_2_ as a solvent, room temperature, 72 h reaction time, Q4 as a catalyst.

### 2.3. Synthesis of Chiral Chalcones Derivatives (−)-**3a**–**i**

The optimized protocol was then expanded to a wide variety of chalcone derivatives and the results are summarized in Table 3. The relationships of the enantioselectivity to different R_1_ and R_2_ values were observed.

**Table 3:** Synthesis of chiral chalcones derivatives (‒)-**3a**–**i** ^[a]^.

Compounds	R_1_	R_2_	Yield [%] ^[b]^	*ee* [%] ^[c]^
(−)-3a	H	8-Me	45.7	81.4
(−)-3b	2,4-diCl	8-Me	40.0	91.5
(−)-3c	H	6-Me	40.0	86.0
(−)-3d	2,4-diCl	6-Me	38.0	91.8
(−)-3e	4-Cl	H	35.0	99.0
(−)-3f	4-MeO	H	42.2	92.0
(−)-3g	4-Cl	6-Me	46.1	95.9
(−)-3h	2-Cl	6-Me	40.3	96.0
(−)-3i	2-F	6-Me	42.5	92.5

^[a]^ Unless otherwise indicated, all reactions were conducted with **2a-i** (0.45 mmol), **1** (0.9 mmol) and the Q4 catalyst (10 mol-%, 0.045 mmol) in CH_2_Cl_2_ (4 mL) at room temperature for 72 h. ^[b]^ Isolated yield after chromatographic purification. ^[c]^ Determined by HPLC analysis (Chiralpak AD-H).

The data indicated that compounds (−)-**3e**, (−)-**3g**, and (−)-**3h** exhibited higher enantioselectivity than other compounds, with *ee* values of 99.0%, 95.9%, and 96.0%, respectively. When R_1_ was H, 2-Cl, 4-Cl or 2-F and R_2_ was H or 6-Me the corresponding target chiral chalcones exhibited excellent enantioselectivity. The strongest enantioselectivity was observed when R_1_ was 4-Cl, 2-Cl and R_2_ was substituted with H, 6-Me- groups. Compared with (−)-**3h**, the target compounds (−)-**3b**, (−)-**3d**, (−)-**3f**, and (−)-**3i** also displayed good enantioselectivity, with *ee* values of 91.5%, 91.8%, 92.0% and 92.5%, respectively. The presence of 4-Cl or 2-Cl atoms in a benzene moiety and 6-Me group or H-atom in a quinazoline ring of chalcones **2** did not significantly affect the reactivity and enantioselectivity of the reaction. An H atom as R_1_ and 6-Me or 8-Me as R_2_ in substrates **2** had some effect on the enantioselectivity of the addition reactions; compounds (−)-**3a** and (−)-**3c** afforded moderate enantioselectivity with *ee* values of 81.4% and 86.0%, respectively.

## 3. Experimental Section

### 3.1. General Information

Unless otherwise stated, all the reagents and reactants were purchased from commercial suppliers; melting points were uncorrected and determined on a XT-4 binocular microscope (Beijing Tech Instrument Co., Beijing, China). The ^1^H-NMR and ^13^C-NMR spectra were recorded on an ECX 500 NMR spectrometer (JEOL, Ltd., Tokoy, Japan) at room temperature operating at 500 MHz for ^1^H-NMR and 125 MHz for ^13^C-NMR, using CDCl_3_, or DMSO-*d*_6_ as solvents and TMS as an internal standard; infrared spectra were recorded in KBr on a VECTOR 22 spectrometer (Bruker Ltd., Ettlingen, German); mass spectral studies were conducted on an Agilent 5973 organic mass spectrometer (Agilent Technologies Inc., Santa Clara, CA, USA). The course of the reactions was monitored by TLC; analytical TLC was performed on silica gel GF_254_ plates; column chromatographic purification was carried out using silicagel. The enantiomeric excess was determined by HPLC using a Chiral pak AD-H column.

### 3.2. Preparation of Chiral Catalyst **Q4**

3-(3,5-Ditrifluoromethylphenylamino)4-methoxybutane-3-en-1,2-dione (1.0 mmol) was slowly added with stirring to a mixture of dichloromethane (4 mL) and 9-aminodeoxyquinine (1.02 mmol). After completion of the addition, the stirring was continued for 12 h at room temperature. The mixture was concentrated, and the crude product was purified by preparative TLC with a mixture of petroleum ether and ethyl acetate (V:V = 1:5) as developing solvent to give chiral catalyst **Q4**. Yield, 56.8%; m.p. 170–171 °C, ${\left[\alpha \right]}_{D}^{25}$ = +70.2 (c = 0.52, DMSO) (lit. [18,19], m.p. 181–183 °C, ${\left[\alpha \right]}_{D}^{25}$ = +65.1 (*c* = 0.55, DMSO)). 

### 3.3. Preparation of Intermediates **2**

4-Hydroxychalcone (3 mmol), 4-chloroquinazoline (3 mmol), K_2_CO_3_ (6.3 mmol), and acetone (15 mL) were added to an oven-dried one-neck 50 mL round-bottom flask equipped with a magnetic stirring bar. The resulting mixture was stirred at 40 °C for 10 h, poured into ice water (40 mL), and then separated. The aqueous phase was acidified with 10% HCl to pH 5–7 and then filtered. The residue was dried and recrystallized from ethanol to obtain compounds **2a**–**i** as white solids [20].

### 3.4. Preparation of Title Chiral Compounds (−)**-3a–3i**

To a well stirred solution of nitromethane (0.9 mmol) and chalcone derivative **2** (0.45 mmol) in 4 mL of dichloromethane, Q4 (0.045 mmol) was added. The resulting mixture was stirred at room temperature and monitored by TLC. After stirring for 72 h, the mixture was concentrated, and the crude product was purified by preparative TLC with a mixture of petroleum ether and ethyl acetate (V:V = 2:1) as developing solvent to give title chiral compounds (−)**-3a**–**3i**.

(−)-*3-(4-((8-Methylquinazolin-4-yl)oxy)phenyl)-4-nitro-1-phenylbutan-1-one * [(−)-**3a**]: Light brown solid; yield 45.7%, m.p. 133–135 °C; ${\left[\alpha \right]}_{D}^{25}$ = −62.4 (c = 1.01, CHCl_3_); IR (KBr, cm^−1^) ν: 3442.8 (C-H, Qu-N=CH-N=C), 1681.3 (C=O), 1610.8 (C=N), 1592.3–1485.4 (C=C, benzene and Qu-ring); ^1^H-NMR (DMSO-*d*_6_, ppm): δ 8.74(s, 1H, Qu-2-H), 8.18 (d, 1H, Qu-7-H, *J* = 9.05 Hz), 7.98 (d, 2H, Ar'-6-H, *J* = 7.45 Hz), 7.89 (d, 1H, Qu-5-H, *J* = 6.85 Hz), 7.66 (t, 2H, Ar-2-H, Ar-6-H, *J*_1_ = 8.00 Hz, *J*_2_ = 7.45 Hz), 7.56–7.51 (m, 4H, Ar'-3-H, Ar'-4-H, Ar'-5-H, Qu-6-H),7.29 (d, 2H, Ar-3-H, Ar-5-H, *J* = 8.60 Hz), 5.06–4.91 (m, 2H, -CH_2_-NO_2_), 4.17–4.11 (m, 1H, CH), 3.69–3.55 (m, 2H, -CH_2_-CO), 2.68 (s, 3H, CH_3_);^13^C-NMR (DMSO-*d*_6_, ppm): δ 197.92, 167.01, 153.57, 151.85, 150.64, 138.08, 136.88, 136.31, 134.92, 134.02, 129.69, 129.32, 128.52, 128.10, 122.60, 121.45, 116.00, 80.18, 41.74, 39.18, 17.68. MS (ESI) *m/z*: 428.3 ([M+H]^+^), 450.3 ([M+Na]^＋^); 81.42% *ee* as determined by HPLC (Daicel Chiralpak AD-H, hexane/ethanol = 50:50, flow rate 1.0 mL/min, λ = 254 nm), t*_R_*(minor) = 33.16 min, t*_R_*(major) = 37.68 min.

(−)-*1-(2,4-Dichlorophenyl)-3-(4-((8-methylquinazolin-4-yl)oxy)phenyl)-4-nitrobutan-1-one* [(−)-**3b**]: Light yellow solid; yield 40.0%, m.p. 65–67°C; ${\left[\alpha \right]}_{D}^{25}$ = −86.4 (c = 0.96, CHCl_3_); IR (KBr, cm^−1^) ν: 3445.2 (C-H, Qu-N=CH-N=C), 1697.4 (C=O), 1614.4 (C=N), 1581.6–1497.4 (C=C, benzene and Qu-ring), 771.53 (C-Cl) cm^−1^; ^1^H-NMR (DMSO-*d*_6_, ppm): δ 8.76( s, 1H, Qu-2-H), 8.19 (d, 1H, Ar'-3-H, *J* = 8.00 Hz), 7.89 (d, 1H, Ar'-3-H, *J* = 7.45 Hz), 7.70 (s, 1H, Qu-7-H), 7.67 (d, 2H, Ar'-5-H, Qu-6-H, *J* = 8.05 Hz), 7.57 (d, 1H, Qu-5-H, *J* = 6.85 Hz), 7.49 (d, 2H, Ar'-2-H, Ar'-6-H, *J* = 8.60 Hz), 7.29 (d, 2H, Ar-3-H, Ar-5-H, *J* = 8.00 Hz), 5.04–4.92 (m, 2H, -CH_2_-NO_2_), 4.08-4.03 (m, 1H, CH), 3.52 (d, 2H, -CH_2_-CO, *J* = 6.85 Hz), 2.69 (s, 3H, CH_3_); ^13^C-NMR (DMSO-*d*_6_, ppm): δ 199.45, 167.06, 153.57, 151.96, 150.71, 137.46, 137.23, 136.83, 136.31, 134.91, 131.68, 131.68, 131.22, 130.64, 129.72, 128.17, 128.10, 122.66, 121.46, 115.92, 79.84, 45.59, 39.29, 17.69. MS (ESI) *m/z*: 496.2 ([M+H]^+^), 518.2 ([M+Na]^+^); 91.45% *ee* as determined by HPLC (Daicel Chiralpak AD-H, hexane/ethanol = 50:50, flow rate 1.0 mL/min, λ = 254 nm), t*_R_*(minor) = 24.49 min, t*_R_*(major) = 27.49 min.

(−)-*3-(4-((6-Methylquinazolin-4-yl)oxy)phenyl)-4-nitro-1-phenylbutan-1-one * [(−)-**3c**]: White solid; yield 40.0%, m.p. 190–102 °C; ${\left[\alpha \right]}_{D}^{25}$ = −78.4 (c = 0.78, CHCl_3_); IR (KBr, cm^−1^) ν: 3450.2 (C-H, Qu-N=CH-N=C), 1685.8 (C=O), 1653.0 (C=N), 1588.1–1498.7 (C=C, benzene and Qu-ring); ^1^H-NMR (DMSO-*d*_6_, ppm): δ 8.66 (s, 1H, Qu-2- H), 8.13 (s, 1H, Qu-4-H), 7.98 (d, 2H, Qu-7-H, Qu-8-H, *J* = 8.05 Hz), 7.91–7.87 (m, 2H, Ar'-2-H, Ar'-6-H), 7.66 (t, 1H, Ar'-4-H, *J*_1_ = 6.90 Hz, *J*_2_ = 7.40 Hz), 7.56–7.52 (m, 4H, Ar'-3-H, Ar'-5-H, Ar-2-H, Ar-6-H), 7.29 (d, 2H, Ar-3-H, Ar-5-H, *J* = 6.90 Hz), 5.06–4.91 (m, 2H, -CH_2_-NO_2_), 4.16–4.10 (m, 1H, CH), 3.68–3.55 (m, 2H, -CH_2_-CO), 2.56 (s, 3H, CH_3_); ^13^C-NMR (DMSO-*d*_6_, ppm): δ 197.46, 166.30, 153.57, 151.81, 150.17, 138.54, 138.06, 137.05, 136.88, 134.02, 129.69, 129.32, 128.51, 127.84, 122.58, 122.53, 115.99, 80.18, 41.74, 39.18, 21.70. MS (ESI) *m/z*: 428.3 ([M+H]^+^), 450.2 ([M+Na]^+^); 86.0% *ee* as determined by HPLC (Daicel Chiralpak AD-H, hexane/ethanol = 50:50, flow rate 1.0 mL/min, λ = 254 nm), t*_R_*(minor) = 8.02 min, t*_R_*(major) = 8.95 min.

(−)**-***1-(2,4-Dichlorophenyl)-3-(4-((6-methylquinazolin-4-yl)oxy)phenyl)-4-nitrobutan-1-one* [(−)-**3d**]: White solid; yield 38.0%, m.p. 142–144 °C; ${\left[\alpha \right]}_{D}^{25}$ = −51.8(c = 0.85, CHCl_3_); IR (KBr, cm^−1^) ν: 3450.0 (C-H, Qu-N=CH-N=C), 1699.3 (C=O), 1653.0 (C=N), 1581.8–1423.5 (C=C, benzene and Qu-ring), 781.2 (C-Cl); ^1^H-NMR (DMSO-*d*_6_, ppm): δ 8.67 (s, 1H, Qu-2-H), 8.14 (s, 1H, Qu-5-H), 7.91–7.86 (m, 2H, Ar'-3-H, Ar'-6-H), 7.74 (d, 1H, Qu-7-H, *J* = 2.30 Hz), 7.67 (d, 1H, Qu-8-H, *J* = 8.05 Hz), 7.58-7.56 (m, 1H, Ar'-5-H), 7.49 (d, 2H, Ar-2-H, Ar-6-H, *J* = 8.55 Hz), 7.29 (d, 2H, Ar-3-H, Ar-5-H, *J* = 8.60 Hz), 5.04–4.92 (m, 2H, -CH_2_-NO_2_), 4.10–4.03 (m, 1H, CH), 3.52 (d, 2H, -CH_2_-CO, *J* = 6.90 Hz); ^13^C-NMR (DMSO-*d*_6_, ppm): δ 199.47, 166.32, 153.57, 151.92, 150.18, 138.54, 137.43, 137.23, 137.04, 131.62, 136.90, 131.20, 130.62, 129.73, 128.16, 127.85, 122.64, 122.55, 116.00, 79.83, 45.60, 39.29, 21.70. MS (ESI) *m/z*: 496.2 ([M+H]^+^), 518.2 ([M+Na]^+^); 91.8% *ee* as determined by HPLC (Daicel Chiralpak AD-H, hexane/ethanol = 50:50, flow rate 1.0 mL/min, λ = 254 nm), t*_R_*(minor) = 6.31 min, t*_R_*(major) = 6.78 min.

(−)-*1-(4-Chlorophenyl)-4-nitro-3-(4-(quinazolin-4-yloxy)phenyl)butan-1-one * [(−)-**3e**]: White solid; yield 35.0%, m.p. 146–148 °C; ${\left[\alpha \right]}_{D}^{25}$ = −56.2 (c = 0.88, CHCl_3_); IR (KBr, cm^−1^) ν: 3450.3 (C-H, Qu-N=CH-N=C), 1683.9 (C=O), 1653.0 (C=N), 1589.3–1489.1 (C=C, benzene and Qu-ring), 700.1 (C-Cl) cm^−1^; ^1^H-NMR (DMSO-*d*_6_, ppm): δ 8.72 (s, 1H, Qu-2-H), 8.35 (d, 1H, Qu-8-H, *J* = 8.00 Hz), 8.04 (t, 1H, Qu-7-H, *J*_1_ = 8.60 Hz, *J*_2_ = 6.85 Hz), 8.00 (t, 3H, Qu-5-H, Ar'-2-H, Ar'-6-H, *J*_1_ = 3.45 Hz, *J*_2_ = 8.60 Hz), 7.79 (t, 1H, Qu-6-H, *J*_1_ = 7.70 Hz, *J*_2_ = 7.15 Hz), 7.61 (d, 2H, Ar'-3-H, Ar'-5-H, *J* = 8.10 Hz), 7.51 (d, 2H, Ar-2-H, Ar-6-H, *J* = 8.60 Hz), 7.30 (d, 2H, Ar-3-H, Ar-5-H, *J* = 8.60 Hz), 5.04–4.89 (m, 2H, -CH_2_-NO_2_), 4.14–4.10 (m, 1H, CH), 3.67–3.55 (m, 2H, -CH_2_-CO); ^13^C-NMR (DMSO-*d*_6_, ppm): δ 197.14, 166.68, 154.43, 151.75, 151.65, 138.99, 138.21, 135.52, 135.26, 130.46, 129.70, 129.43, 128.67, 128.05, 123.93, 122.63, 116.16, 80.13, 41.75, 39.12. MS (ESI) *m/z*: 448.2 ([M+H]^+^), 470.2 ([M+Na]^+^); 99% *ee* as determined by HPLC (Daicel Chiralpak AD-H, hexane/ethanol = 50:50, flow rate 1.0 mL/min, λ = 254 nm), t*_R_*(major) = 9.61 min.

(−)**-***1-(4-Methoxyphenyl)-4-nitro-3-(4-(quinazolin-4-yloxy)phenyl)butan-1-one * [(−)-**3f**]: White solid; yield 42.2%, m.p. 144–146 °C; ${\left[\alpha \right]}_{D}^{25}$ = −49.8 (c = 0.88, CHCl_3_); IR (KBr, cm^−1^) ν: 3450.3 (C-H, Qu-N=CH-N=C), 1683.9 (C=O), 1653.0 (C=N), 1589.3-1489.1 (C=C, benzene and Qu-ring);^1^H-NMR (DMSO-*d*_6_, ppm): δ 8.73 (s, 1H, Qu-2-H), 8.35 (d, 1H, Qu-8-H, *J* = 7.40 Hz), 8.03 (t, 1H, Qu-7-H, *J*_1_ = 8.60 Hz, *J*_2_ = 6.85 Hz), 8.00 (d, 1H, Qu-5-H, *J* = 8.05 Hz), 7.96 (t, 1H, Qu-6-H, *J*_1_ = 8.00 Hz, *J*_2_ = 6.85 Hz), 7.51 (d, 2H, Ar'-3-H, Ar'-5-H, *J* = 8.60 Hz), 7.30 (d, 2H, Ar-2-H, Ar-6-H, *J* = 8.60 Hz), 7.05 (d, 2H, Ar-3-H, Ar-5-H, *J* = 8.60 Hz), 5.05–4.90 (m, 2H, -CH_2_-NO_2_), 4.15–4.09 (m, 1H, CH), 3.85 (s, 3H, CH_3_), 3.61–3.46 (m, 2H, -CH_2_-CO); ^13^C-NMR (DMSO-*d*_6_, ppm): δ 196.21, 166.80, 154.44, 151.71, 151.62, 138.99, 138.21, 135.52, 135.26, 130.46, 129.70, 129.43, 128.67, 128.05, 123.93, 122.63, 116.16, 80.13, 41.75, 39.12. MS (ESI) *m/z*: 444.3 ([M+H]^+^), 466.2 ([M+Na]^+^); 92.0% *ee* as determined by HPLC (Daicel Chiralpak AD-H, hexane/ethanol = 50:50, flow rate 1.0 mL/min, λ = 254 nm), t*_R_*(minor) = 14.50 min, t*_R_*(major) = 15.20 min.

(−)-*1-(4-Chlorophenyl)-3-(4-((6-methylquinazolin-4-yl)oxy)phenyl)-4-nitrobutan-1-one* [(−)-**3g**]:White solid; yield 46.1%, m.p. 210–212 °C; ${\left[\alpha \right]}_{D}^{25}$ = −9.8 (c = 1.20, CHCl_3_); IR (KBr, cm^−1^) ν: 3444.9 (C-H, Qu-N=CH-N=C), 1678.1 (C=O), 1589.3 (C=N), 1548.9–1496.8 (C=C, benzene and Qu-ring), 814.0 (C-Cl); ^1^H-NMR (DMSO-*d*_6_, ppm): δ 8.66 (s, 1H, Qu-2-H), 8.14 (s, 1H, Qu-5-H), 8.00 (d, 2H, Ar'-2-H, Ar'-6-H, *J* = 4.00 Hz), 7.88 (s, 2H, Qu-7-H, Qu-8-H), 7.62 (d, 2H, Ar'-3-H, Ar'-5-H, *J* = 12.60 Hz), 7.51 (d, 2H, Ar-2-H, Ar-6-H, *J* = 4.55 Hz), 7.28 (d, 2H, Ar-3-H, Ar-5-H, *J* = 4.60 Hz), 5.04–4.90 (m, 2H, -CH_2_-NO_2_), 4.17–4.08 (m, 1H, CH), 3.67–3.55 (m, 2H, -CH_2_-CO), 2.57 (s, 3H, CH_3_); ^13^C-NMR (DMSO-*d*_6_, ppm): δ 197.39, 166.39, 153.57, 151.82, 150.16, 138.99, 138.54 , 137.87, 137.08, 135.56, 130.80, 129.19, 127.84, 122.65, 122.53, 120.06, 116.13, 79.68, 42.01, 39.54, 21.71. MS (ESI) *m/z*: 462.2 ([M+H]^+^), 484.2 ([M+Na]^+^); 95.9% *ee* as determined by HPLC (Daicel Chiralpak AD-H, hexane/ethanol = 50:50, flow rate 1.0 mL/min, λ = 254 nm), t*_R_*(minor) = 9.15 min, t*_R_*(major) = 10.43 min.

(−)-*1-(2-Chlorophenyl)-3-(4-((6-methylquinazolin-4-yl)oxy)phenyl)-4-nitrobutan-1-one* [(−)-**3h**]: White solid; yield 40.3%, m.p. 67–69 °C; ${\left[\alpha \right]}_{D}^{25}$ = −32.8.4 (c = 0.97, CHCl_3_); IR (KBr, cm^−1^) ν: 3442.9 (C-H, Qu-N=CH-N=C), 1699.3 (C=O), 1653.0 (C=N), 1580.5–1498.7 (C=C, benzene and Qu-ring), 752.2 (C-Cl) cm^−1^; ^1^H-NMR (DMSO-*d*_6_, ppm): δ 8.67 (s, 1H, Qu-2-H), 8.14 (s, 1H, Qu-5-H), 7.92–7.87 (m, 2H, Qu-6-H, Ar'-6-H), 7.59 (d, 1H, Qu-7-H, *J* = 8.05 Hz), 7.56–7.53 (m, 2H, Ar'-3-H, Ar'-5-H), 7.49 (d, 2H, Ar-2-H, Ar-6-H, *J* = 8.60 Hz), 7.47–7.44 (m, 1H, Ar'-4-H), 7.29 (d, 2H, Ar-3-H, Ar-5-H, *J* = 8.60 Hz), 5.05–4.93 (m, 2H, -CH_2_-NO_2_), 4.11–4.05 (m, 1H, CH), 3.57–3.47 (m, 2H, -CH_2_-CO), 2.57 (s, 3H, CH_3_);^13^C-NMR (DMSO-*d*_6_, ppm): δ 200.48, 166.39, 153.58, 152.02, 150.18, 138.76, 138.55, 137.48, 137.05, 133.01, 131.02, 130.12, 129.74, 129.61, 127.96, 127.86, 122.64, 122.64, 116.00, 79.85, 45.72, 39.31, 21.70. MS (ESI) *m/z*: 462.2 ([M+H]^+^), 484.2 ([M+Na]^+^); 96.0% *ee* as determined by HPLC (Daicel Chiralpak AD-H, hexane/ethanol = 50:50, flow rate 1.0 mL/min, λ = 254 nm), t*_R_*(minor) = 29.56 min, t*_R_*(major) = 31.80 min.

(−)-*1-(2-Fluorophenyl)-3-(4-((8-methylquinazolin-4-yl)oxy)phenyl)-4-nitrobutan-1-one* [(−)-**3i**]:White solid; yield 42.5%, m.p. 123–125 °C; ${\left[\alpha \right]}_{D}^{25}$ = −43.9 (c = 0.82, CHCl_3_); IR (KBr, cm^−1^) ν: 3442.9 (C-H, Qu-N=CH-N=C), 1678.1 (C=O), 1608.6 (C=N), 1545.0–1483.3 (C=C, benzene and Qu-ring); ^1^H-NMR (DMSO-*d*_6_, ppm): δ 8.76 (s, 1H, Qu-2-H), 8.18 (d, 1H, Qu-5-H, *J* = 8.00 Hz), 7.89 (d, 1H, Ar'-6-H, *J* = 7.45 Hz), 7.82 (t, 1H, Ar'-4-H, *J*_1_ = 7.40 Hz, *J*_2_ = 7.45 Hz), 7.70–7.65 (m, 2H, Qu-7-H, Qu-8-H), 7.51 (d, 2H, Ar-2-H, Ar-6-H, *J* = 8.55 Hz), 7.39–7.33 (m, 2H, Ar'-3-H, Ar'-5-H), 7.30 (d, 2H, Ar-3-H, Ar-5-H, *J* = 8.05 Hz), 5.07–4.94 (m, 2H, -CH_2_-NO_2_), 4.16–4.11 (m, 1H, CH), 3.54 (d, 2H, -CH_2_-CO, *J* = 5.15 Hz), 2.69 (s, 3H, CH_3_); ^13^C-NMR (DMSO-*d*_6_, ppm): δ 195.83, 167.00, 160.78, 153.57, 151.85, 150.63, 138.01, 136.31, 135.92, 134.91, 130.80, 129.65, 128.10, 125.40, 122.64, 121.45, 117.58, 117.40, 116.00, 79.99, 46.21, 39.03, 17.69. MS (ESI) *m/z*: 446.3 ([M+H]^+^), 484.2 ([M+Na]^+^); 92.5% *ee* as determined by HPLC (Daicel Chiralpak AD-H, hexane/ethanol = 50:50, flow rate 1.0 mL/min, λ = 254 nm), t*_R_*(minor) = 27.99 min, t*_R_*(major) = 29.44 min.

## 4. Conclusions

In summary, we have developed and reported for the first time an efficient approach for enantioselective synthesis of (−)-1-phenyl-3-(4-((8-methylquinazolin-4-yl)oxy)phenyl)-4-nitrobutan-1-ones by employing an addition reaction catalyzed by a cinchona alkaloid-derived squaramide catalyst. The desired products were obtained with high enantioselectivities (81%–99%).
